# Ablation of T cell–associated PD-1H enhances functionality and promotes adoptive immunotherapy

**DOI:** 10.1172/jci.insight.148247

**Published:** 2022-01-25

**Authors:** Li Hu, Ling Chen, Zexiu Xiao, Xu Zheng, Yuangui Chen, Na Xian, Christina Cho, Liqun Luo, Gangxiong Huang, Lieping Chen

**Affiliations:** 1 Institute of Immunotherapy, Fujian Medical University, Fuzhou, Fujian, China.; 2 Laboratory of Immunotherapy, Sun Yat-sen University, Guangzhou, Guangdong, China.; 3 Department of Radiation Oncology, Fujian Medical University Union Hospital, Fuzhou, Fujian, China.; 4 Tcelltech Biological Science and Technology Inc., Fuzhou, Fujian, China.; 5 Department of Immunobiology, Yale University, New Haven, Connecticut, USA.

**Keywords:** Immunology, Therapeutics, Cancer immunotherapy, T cells

## Abstract

Programmed death-1 homolog (PD-1H) is a coinhibitory molecule that negatively regulates T cell–mediated immune responses. In this study, we determined whether ablation of T cell–associated PD-1H could enhance adoptive T cell therapy in experimental tumor models. The expression of PD-1H is upregulated in activated and tumor-infiltrating CD8^+^ T cells. Activated CD8^+^ T cells from PD-1H–deficient (PD-1H–KO) mice exhibited increased cell proliferation, cytokine production, and antitumor activity in vitro. Adoptive transfer of PD-1H–KO CD8^+^ T cells resulted in the regression of established syngeneic mouse tumors. Similar results were obtained when PD-1H was ablated in T cells by CRISPR/Cas9-mediated gene silencing. Furthermore, ablation of PD-1H in CAR-T cells significantly improved their antitumor activity against human xenografts in vivo. Our results indicate that T cell–associated PD-1H could suppress immunity in the tumor microenvironment and that targeting PD-1H may improve T cell adoptive immunotherapy.

## Introduction

Programmed death-1 homolog (PD-1H, also called VISTA, VSIR, Dies1, Gi24, DD1α) is a member of the B7-CD28 family of cell membrane proteins (1, 2). While PD-1H shares sequence homology with several molecules in the gene family such as PD-1 and B7-H1, its structure is unique, implying different functions (3). Mouse PD-1H expression is largely restricted to hematopoietic cells and is constitutively expressed on myeloid cells (macrophages, DCs, monocytes, and neutrophils) in low levels on T lymphocytes, whereas B cells and NK cells are mostly negative (2). Similarly, PD-1H is largely undetectable in freshly isolated human T cells (4). Cell surface PD-1H can be further upregulated by activation of T cells; however, this expression is quickly lost in cell culture without clear reason (2, 4).

Ample evidence supports that PD-1H is a negative immune regulatory protein. The studies showed that PD-1H–KO (PK) mice had increased T cell–mediated immune responses to various antigens compared with PD-1H competent mice (5–8). Despite an overall hyperresponsive to antigen stimulation, there was no obvious autoimmune phenotypes developed in PK mice (5, 7), implying that PD-1H is a molecule involved in fine-tuning of immune responses but not a major protein in the suppression of autoimmunity. Consistent to these findings, a recent study reveals that PD-1H regulates quiescence of CD4^+^ T cells (9). T cell–associated PD-1H has been shown to be a coinhibitory receptor to regulate T cell tolerance and immunity (5), while PD-1H has also been shown to function as a ligand in antigen-presenting cells (APCs) to suppress T cell proliferation and cytokine production (2). Several attempts to identify the counter receptor of PD-1H have revealed multiple binding partners, while their functions are less clear (10). Interestingly, a recent study shows that PD-1H binds to P-selectin glycoprotein ligand 1 (PSGL-1) and subsequently suppresses T cell activation in an acidic environment that occurs in the tumor microenvironment (TME) (11). Blocking PD-1H with a specific antagonist mAb can significantly reduce tumor growth in multiple mouse tumor models, including B16 melanoma, MB49 bladder carcinoma, and PTEN/BRAF inducible melanoma by promoting T cell–mediated immunity (12). In contrast, treatment with PD-1H agonistic mAbs reduced severity of autoimmune diseases in the mouse models of lupus (13), asthma (14, 15), and arthritis (16).

In the context of PD-1H’s role as a T cell regulatory molecule, we explore the possibility of eliminating or decreasing PD-1H expression on CD8^+^ T cells as approaches to promote their functionalities and to enhance adoptive immunotherapy in mouse tumor models.

## Results

### Cell surface PD-1H is upregulated and maintained on activated T cells.

**Figure 1:**
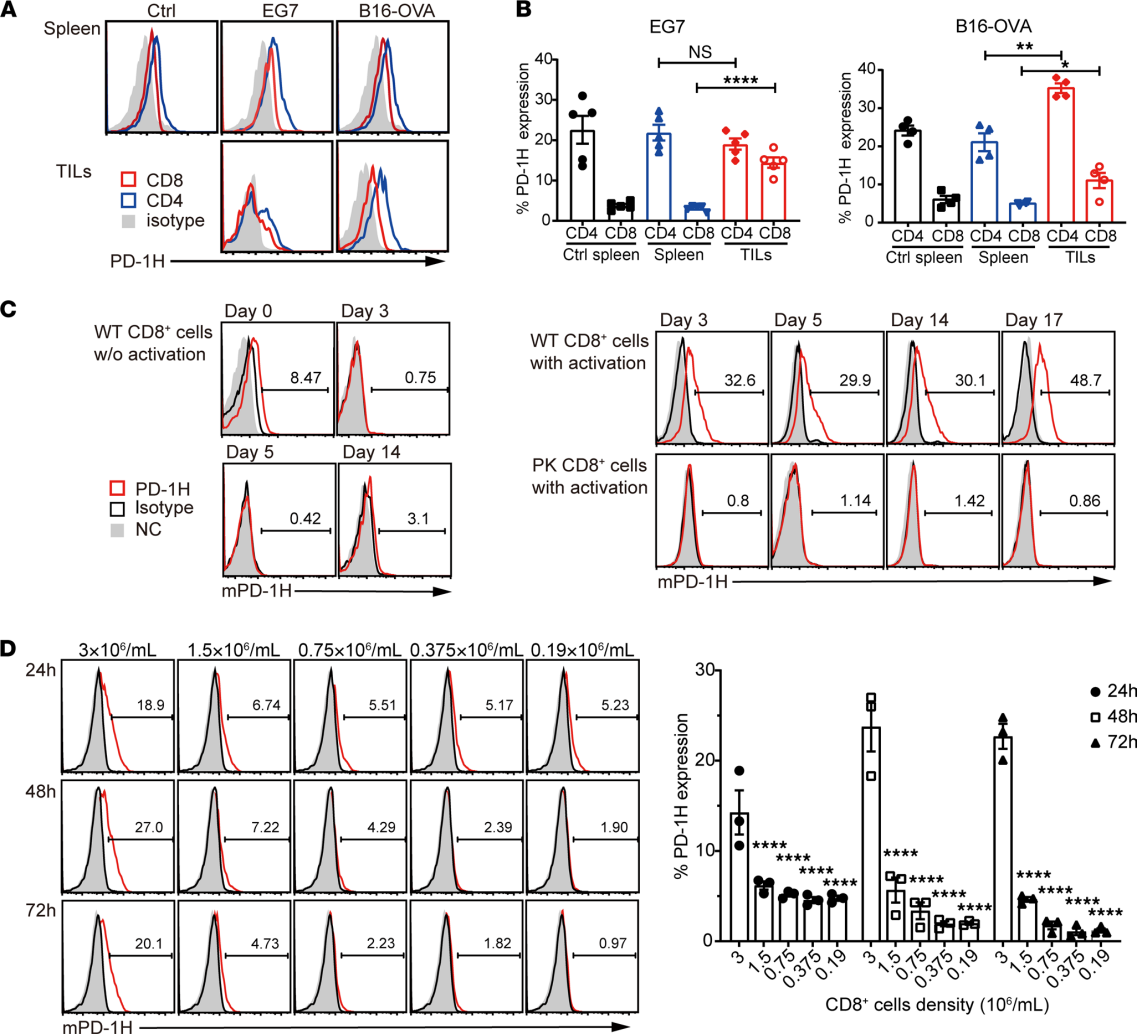
Upregulation of PD-1H expression is maintained on activated T cells. (**A** and **B**) Tumor cells were inoculated into the flank of WT mice (*n* = 4 for B16-OVA; *n* = 5 for EG7 group). Tumors were harvested when the tumor reaches 10–15 mm. Expression of PD-1H on spleen and TILs, including CD4^+^ and CD8^+^ T cells, was analyzed by flow cytometry. Data are representative of 3 independent experiments with mean ± SEM. *P < 0.05, **P < 0.01, and ****P < 0.0001 (2-tailed unpaired *t* test). (**C**) The percentage of PD-1H expression on CD8^+^ T cells. WT or PK CD8^+^ T cells were either freshly isolated and in vitro culture for 3, 5, 14 days, with and without activation (1 μg/mL anti-mCD3 plus 2 μg/mL anti-mCD28). The expression of PD-1H on CD8^+^ T cells was analyzed by flow cytometry at days 3, 5, 14, and 17. (**D**) In total, 3 × 10^6^/mL activated PD-1H^+^ CD8^+^ T cells were diluted in culture in vitro in a 96 well-plate; PD-1H expression was detected at 24, 48, and 72 hours by flow cytometry. Data are presented as mean ± SEM and represent 3 independent experiments. ****P < 0.0001 (2-way ANOVA).

To determine the potential role of PD-1H on CD8^+^ T cells, we first evaluated the expression of PD-1H in tumor-infiltrating T lymphocytes (TILs) in the EG7 lymphoma and B16-OVA melanoma mouse models. Seventeen days after tumor cell inoculation, TILs were isolated and analyzed for PD-1H expression by flow cytometry. Splenic T cells from tumor-bearing and normal mice were used as controls. Both TILs and splenic T lymphocytes expressed PD-1H on the cell surface, with CD4^+^ T cells having higher levels of expression than CD8^+^ T cells (Figure 1A). Interestingly, PD-1H was significantly upregulated in both CD4^+^ and CD8^+^ T cells in B16-OVA melanoma tumors compared with splenic T cells. The similar upregulation of PD-1H was also observed in CD8^+^ but not in CD4^+^ T cells in EG7 lymphoma tumors (Figure 1B and Supplemental Figure 1A; supplemental material available online with this article; https://doi.org/10.1172/jci.insight.148247DS1). These results suggest that PD-1H may be induced in antigen-activated T cells. CD8^+^ T cells express lower levels of PD-1H in their inactivated status compared with CD4^+^ T cells, and this may be attributed to the fact that PD-1H is more prone to being upregulated in CD8^+^ T cells.

Expression status of PD-1H in activated CD8^+^ T cells was evaluated. CD8^+^ T cells were freshly isolated from WT or PK mice and cultured in medium with or without anti-CD3 antibody. In contrast to earlier reports (2), PD-1H was upregulated in activated WT CD8^+^ T cells throughout cell culture (days 3–17) (Figure 1C). To assess whether maintenance of cell surface PD-1H expression was dependent on cell density, we performed a serial dilution experiment. By 24 hours, PD-1H expression was lost on CD8^+^ T cells that were seeded at low density (less than 3 × 10^6^/mL). In contrast, PD-1H expression was maintained on cells seeded at a higher density even after 72 hours in culture (Figure 1D and Supplemental Figure 1B). These results indicate that PD-1H expression is dependent on cell density; thus, it is subject to variation from experiment to experiment and may explain the contradiction between our findings and those previously reported.

Altogether, these results indicate that PD-1H expression is inducible in CD8^+^ T cells and that this induction may be triggered by T cell–activating signals. Given that PD-1H is a coinhibitory molecule that it is upregulated upon T cell activation, we hypothesize that PD-1H induction in tumor-infiltrating CD8^+^ T cells alters their function within the TME.

### Downregulation of PD-1H enhances the antitumor activity of CD8^+^ T cells.

The role of PD-1H in the antitumor activity of CD8^+^ T cells was evaluated in vivo using syngeneic mouse tumor models. WT and PK mice were inoculated with B16-OVA melanoma cells, and tumor growth was monitored over time. Although primary tumor growth was similar between WT and PK mice, the percentage of myeloid-derived suppressor cells (MDSCs) within the TME was reduced in PK mice compared with WT mice (Supplemental Figure 1C). These results suggest that loss of PD-1H in MDSCs has no direct effect in controlling tumor growth, despite the fact that MDSCs are a major component of tumor-infiltrating cells and are known to express high levels of PD-1H (12, 17).

**Figure 2:**
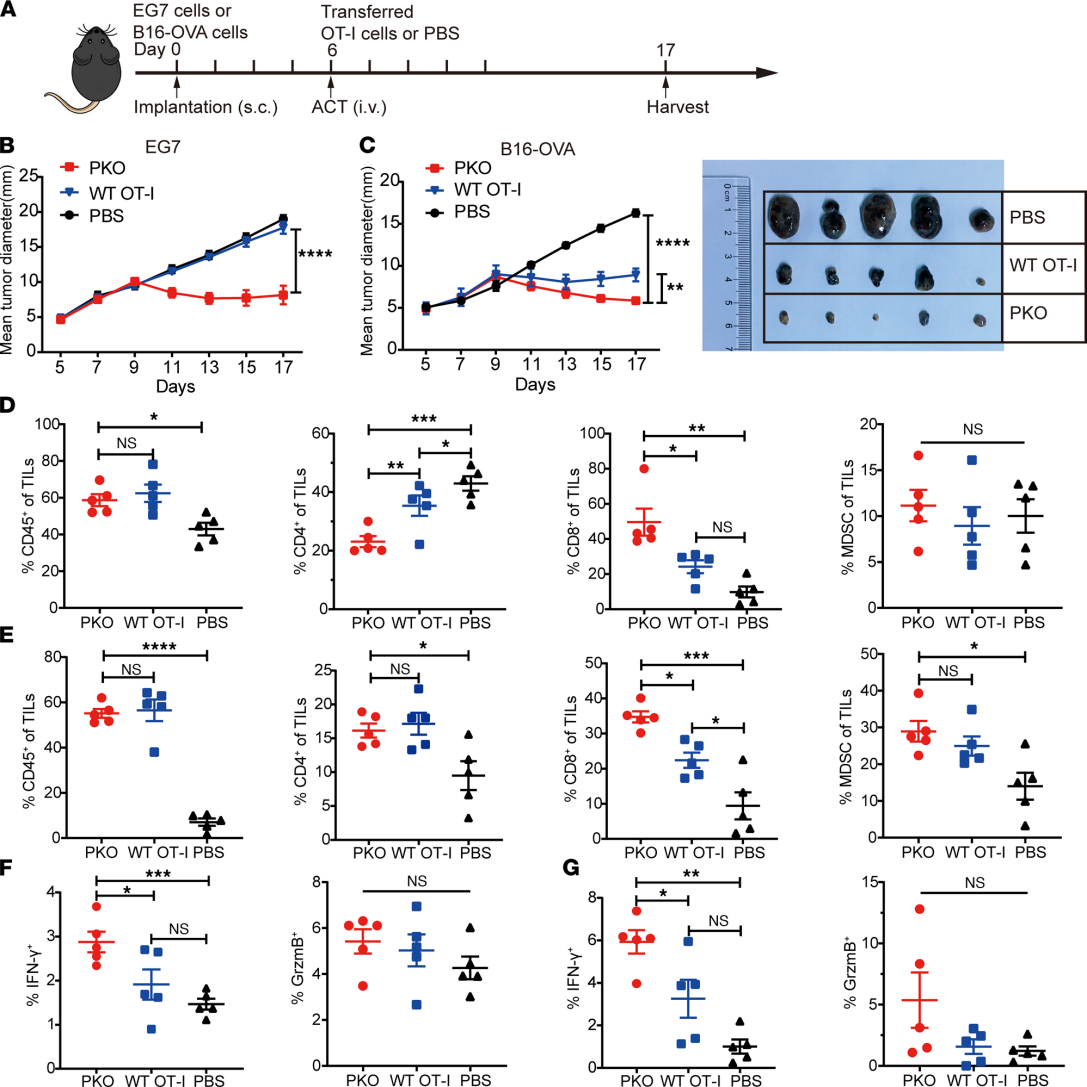
PKO cell treatment suppressed tumor growth in mouse tumor model. (**A**) EG7 or B16-OVA tumor–bearing mice (*n* = 5 per group) received an i.v. injection of OT-I cells or PBS on day 6. (**B** and **C**) Tumor growth was measured by caliper every 2 days. Data are representative of 3 independent experiments with mean ± SEM. **P < 0.01, ****P < 0.0001 (2-way ANOVA). Tumors were harvested, and CD45^+^ TILs were analyzed on day 17. (**D** and **E**) TILs including CD4^+^ and CD8^+^ T cells, and MDSCs (CD11b^+^CD11c^–^Gr-1^+^) were identified by flow cytometry (EG7 [**D**] and B16-OVA [**E**]). (**F** and **G**) The expression level of effector molecules (IFN-γ and granzyme B) by TILs was analyzed after 5 hours of stimulation with OVA peptide (EG7 [**F**] and B16-OVA [**G**]). Data are representative of 2 independent experiments with mean ± SEM. *P < 0.05, **P < 0.01, ***P < 0.001, and ****P < 0.0001 (1-way ANOVA).

Since PD-1H is upregulated in tumor-infiltrating CD8^+^ T cells (Figure 1), we evaluated the role of PD-1H in CD8^+^ T cell function in established tumors in an adoptive cell therapy model. We generated PK OT-I (PKO) mice by backcrossing PK mice with OT-I TCR transgenic mice. CD8^+^ T cells from WT OT-I and PKO mice were then transferred i.v. into WT mice 6 days after inoculation with EG7 cells or B16-OVA cells (Figure 2). While EG7 tumor growth was similar between control mice and mice that received WT OT-I cells in this given dose, tumor growth was markedly reduced in mice that received PKO cells (Figure 2B). Likewise, maximal suppression of B16-OVA tumor growth was observed in mice that received PKO cells (Figure 2C). This suggested that delayed tumor growth may be due to increased infiltration and function of PKO cells.

To determine whether loss of PD-1H enhances CD8^+^ T cell tumor infiltration, we analyzed the frequency of T cell populations within the TME. Compared with tumors generated in control mice, tumors established in WT OT-I– and PKO-transferred mice contained significantly higher percentages of CD45^+^ T cells. Further analysis revealed that the frequency of CD8^+^ T cells was highest in PKO cell-transferred mice, whereas CD4^+^ T cells were enriched in WT OT-I–transferred mice. There were no differences in the percentage of MDSCs between T cell–transferred groups (Figure 2, D and E). Thus, these data indicate that PD-1H downregulation may improve CD8^+^ T cell infiltration into the tumor.

Next, we evaluated the role of PD-1H in CD8^+^ T cell function within the TME. Isolated TILs were simulated with OVA peptides ex vivo, and then IFN-γ and granzyme B production was assessed. The percentage of IFN-γ^+^ cells in tumors generated in the PKO cells–transferred group was significantly higher than the WT OT-I cells–transferred group, whereas there was no difference in granzyme B production between groups (Figure 2, F and G). Altogether, these data suggest that loss of PD-1H enhances the activity of tumor-infiltrating CD8^+^ T cells and results in the regression of established solid tumors.

### T cell–associated PD-1H suppresses the function of CD8^+^ T effector cells ex vivo.

**Figure 3:**
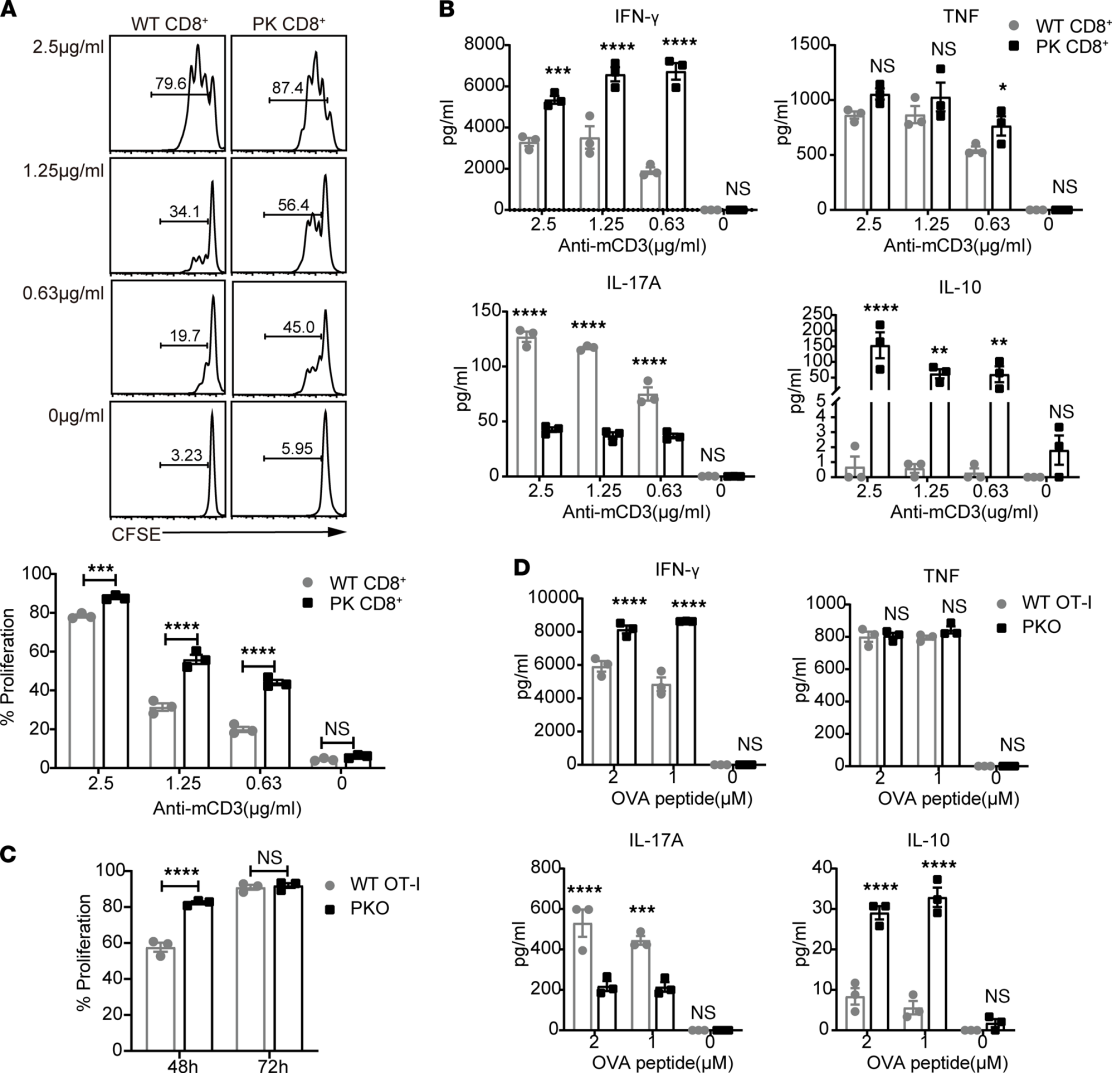
PD-1H suppresses the function of CD8^+^ T cells ex vivo. CFSE-labeled WT or PK CD8^+^ T cells were stimulated by plate-bound anti-mCD3. (**A**) Cell proliferation was analyzed at the end of 72 hours of culture. (**B**) Supernatants were harvested from 96-well proliferation plates at 72 hours and analyzed for IFN-γ, IL-17A, and TNF. (**C**) Purified of PKO or WT OT-I cells were stimulated with 1 μM OVA peptide–pulsed irradiated PK splenocytes. Proliferation was analyzed at 48 and 72 hours. (**D**) Supernatants were harvested from proliferation assays at 48 hours and analyzed for cytokines at specific peptide concentrations. Duplicated wells were analyzed for all conditions. All data are mean ± SEM and represent 3 independent experiments. *P < 0.05; **P < 0.01; ***P < 0.001; ****P < 0.0001 by 2-way ANOVA.

To further assess whether loss of PD-1H in CD8^+^ T cells enhances their function, naive T cells — isolated from PK and WT mice — were activated ex vivo using an anti-CD3 mAb, and then proliferation and production of various cytokines was assessed. Activated PK CD8^+^ T cells exhibited higher proliferative capacity in vitro than WT CD8^+^ T cells (Figure 3A). In addition, PK CD8^+^ T cells produced increased levels of IFN-γ and IL-10, and they produced decreased levels of IL-17A compared with WT CD8^+^ T cells, while no difference was observed in TNF production between the 2 cell types (Figure 3B). These results were verified by experiments involving an alternative approach in which WT OT-I and PKO cells were activated by coculture with OVA peptide–pulsed PK APCs (Figure 3C). Compared with OVA-activated WT OT-I cells, OVA-activated PKO cells exhibited enhanced proliferation and stronger cytokine production (IFN-γ and IL-10) (Figure 3D). In addition, PK T cells expressed slightly higher–level memory markers. WT OT-I cells appear to have higher levels of PD-1, Tim3, and 2B4 than PKO cells. The expression of Bcl-2, T-bet, Eomes, and TOX showed no significant differences between each other (Supplemental Figure 2).

**Figure 4:**
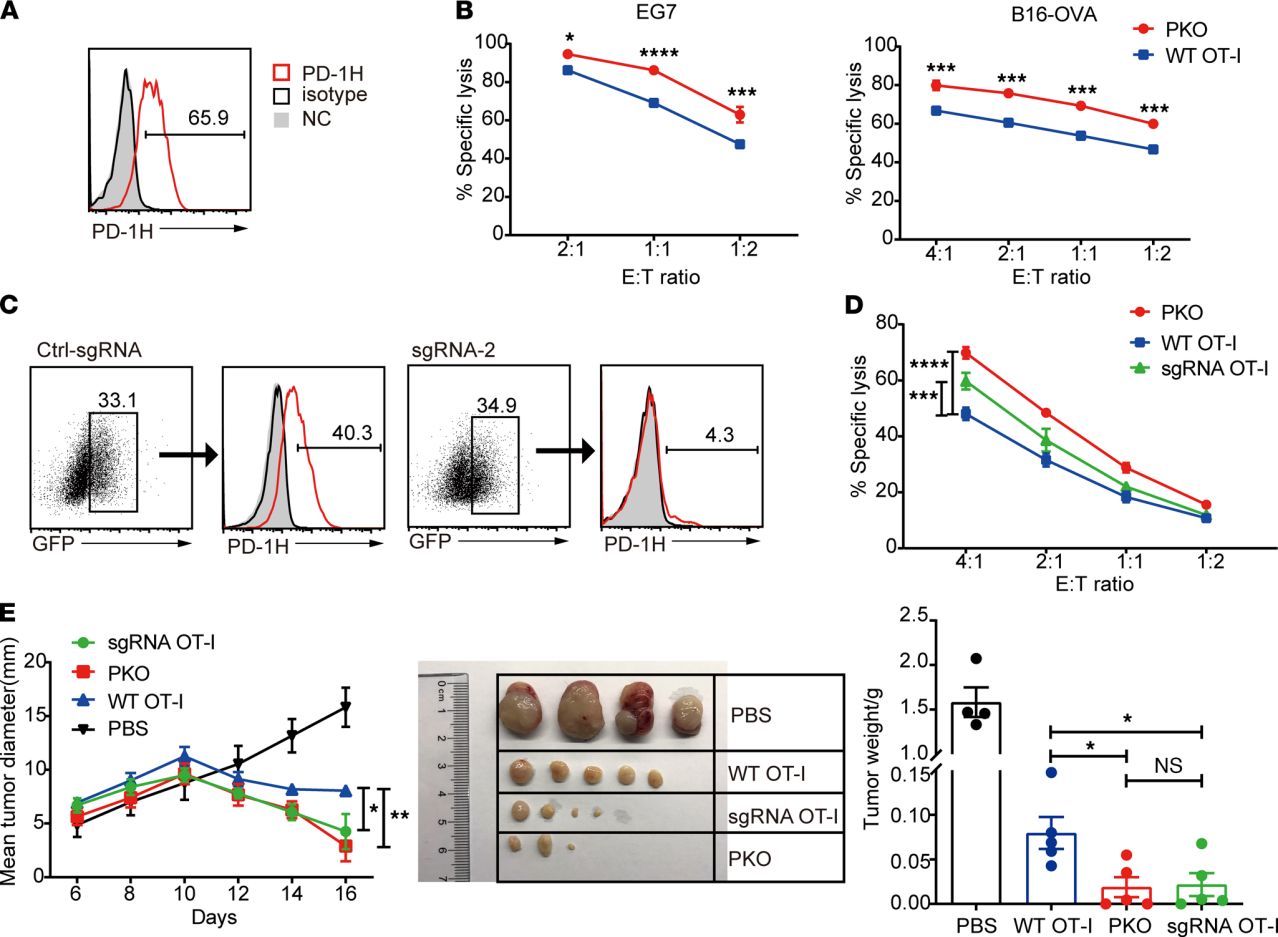
PD-1H ablation enhances the effector function of CD8^+^ T cells. (**A**) The expression of PD-1H on activated WT OT-I cells was analyzed by flow cytometry. (**B**) CFSE-labeled EG7 or B16-OVA tumor cells were coincubated with activated PKO or WT OT-I cells at the different effector/target (E:T) ratios for 4 hours. Cells were stained with DAPI and analyzed by flow cytometry. Data are representative of 3 independent experiments with mean ± SEM. *P < 0.05, ***P < 0.001, and ****P < 0.0001 (2-way ANOVA). (**C**) CRISPR/Cas9 efficiently disrupted the expression of PD-1H on CD8^+^ T cells. PD-1H expression was analyzed in the GFP^+^ gate by flow cytometry. (**D**) The killing activity of sgRNA OT-I cells on EG7 cells in vitro was analyzed by CFSE/DAPI staining. EG7-bearing mice (*n* = 4 for the control group; *n* = 5 per group) received an i.v. injection of OT-I cells or PBS. (**E**) Tumor growth was monitored from day 6, and tumor weight at day 16 is shown. NC, negative control; Ctrl, control. Data are representative of 2 independent experiments with mean ± SEM. *P* values by 2-tailed unpaired *t* test (right graph in **E**) and 2-way ANOVA (left graph in **E**). *P < 0.05; **P < 0.01; ***P < 0.001; ****P < 0.0001.

We also evaluated the effect of endogenous PD-1H on T cell activity using an in vitro cytotoxicity assay. First, we confirmed the constitutive expression of PD-1H on activated WT OT-I cells by flow cytometry (Figure 4A). Next, we coincubated activated WT OT-I or PKO cells with EG7 or B16-OVA tumor cells for 4 hours and evaluated the percentage of lysed tumor cells. Compared with PKO cells, WT OT-I cells exhibited significantly reduced cytotoxic function (Figure 4B) and production of IFN-γ and TNF-α (Supplemental Figure 3). These results support our hypothesis that PD-1H functions as a coinhibitory receptor to suppress the cytotoxic function of CD8^+^ T cells.

### Gene editing-mediated PD-1H ablation enhances the effector function of CD8^+^ T cells.

Since downregulation of PD-1H enhances the tumoricidal activity of CD8^+^ T cells (Figure 4B), disruption of intrinsic PD-1H in cytotoxic T lymphocytes (CTLs) may improve the efficacy of cell-based immunotherapies. To determine which system would most efficiently downregulate PD-1H in CTLs, we first compared the efficiency of PD-1 knockdown by CRISPR/Cas9 and shRNA interference in CHO cells that stably express mouse PD-1 (CHO/mPD-1). Although both systems reduced PD-1 expression in these cells, the CRISPR/Cas9 system was appreciably more efficient (Supplemental Figure 4). Thus, we decided to utilize the CRISPR/Cas9 system for our investigations.

Two sgRNAs targeting the second exon of PD-1H were designed, and their gene editing efficiencies in NIH/3T3 cells were evaluated using the T7EN1 cleavage assay and tracking of indels by decomposition (TIDE) analysis (18). The knockdown efficiency of sgRNA-2 was greater than that of sgRNA-1 in NIH/3T3 cells (Supplemental Figure 5A; see complete unedited blots in the supplemental material); thus, we decided to continue our studies using only sgRNA-2. DNA sequence, mRNA, protein, and off-target analyses were examined to confirm the efficiency and specificity of sgRNA-2 in PD-1H targeting (Supplemental Figure 5, B–D). Next, we delivered CRISPR/Cas9 gene editing components guided by sgRNA-2 into mouse CD8^+^ T cells using a lentiviral vector system. The percentage of GFP^+^CD8^+^ T cells indicated the efficiency of lentiviral transduction (Supplemental Figure 5E). Three days after transduction, gene editing efficiency was confirmed by T7EN1 cleavage assay and by sequencing the mutation at the genomic locus of PD-1H (Supplemental Figure 5E). This CRISPR/Cas9 system guided by sgRNA-2 was therefore used to generate PK OT-I cells (sgRNA OT-I cells).

Seven days after transducing OT-I T cells with sgRNA-2–guided Cas9, PD-1H expression was determined by flow cytometry (Figure 4C). Next, effector T cells — WT OT-I, PKO, or sgRNA OT-I cells — were cocultured with CFSE-labeled EG7 tumor cells for 4 hours, and the killing activity of each group of T cells was determined using flow cytometry. Compared with WT OT-I cells, sgRNA OT-I cells exhibited superior cytolytic ability on EG7 tumor cells in a dose-dependent manner (Figure 4D). As expected, PKO cells exhibited the highest cytolytic activity because of a complete ablation of PD-1H. Additionally, we assessed the antitumor activity of these effector cells using a therapeutic model of adoptive T cell transfer. WT OT-I, PKO, or sgRNA OT-I cells were transferred into mice bearing EG7 tumors, and changes in tumor growth were evaluated. We found that both sgRNA OT-I and PKO cells were significantly more effective in suppressing tumor growth than WT OT-I cells (Figure 4E). Altogether, these results show that inactivation of intrinsic PD-1H by gene editing can promote the antitumor activity of CD8^+^ T cells.

### PD-1H regulates the chemotactic activity of adoptive CD8^+^ T cells.

**Figure 5:**
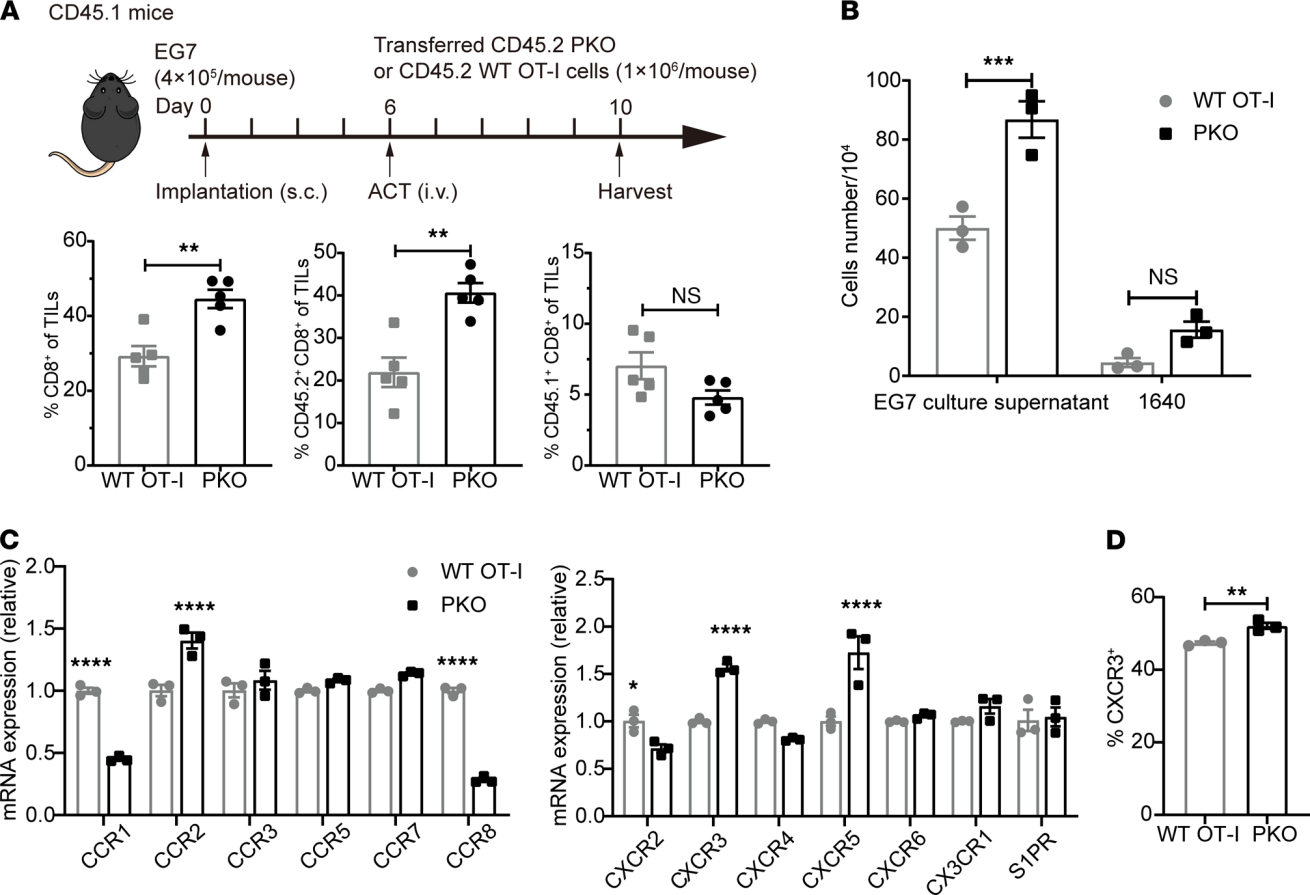
PD-1H regulates the chemotactic capabilities of CD8^+^ T cells. (**A**) CD45.1^+^ congenic mice were inoculated with EG7 tumors and were i.v. injected with purified CD45.2^+^ WT OT-I or PKO cells on day 6 (*n* = 5 per group). Tumor was harvested and analyzed on day 10. The percentage of total CD8^+^ TILs from each group is shown on the left bar graph. The middle bar graph shows the percentage of donor CD45.2^+^CD8^+^ TILs, and the right bar graph shows the percentage of host CD45.1^+^CD8^+^ TILs from each mouse. Data are representative of 2 independent experiments with mean ± SEM. **P < 0.01 (2-tailed unpaired *t* test). (**B**) Activated WT OT-I or PKO cells were placed in upper wells of 5 μm transwell plates and allowed to migrate to the lower chamber containing EG7 culture supernatant or 1640 medium for 3.5 hours. Cells that had migrated to the bottom chamber were counted. Data are mean ± SEM and represent 3 independent experiments. ***P < 0.001 (2-way ANOVA). (**C**) WT OT-I or PKO cells were collected to quantify mRNA expression of chemokine receptor by real-time PCR. (**D**) The expression of CXCR3 was analyzed by flow cytometry. Data are mean ± SEM and represent 3 independent experiments. *P < 0.05, **P < 0.01, ****P < 0.0001 by 2-tailed unpaired *t* test and 2-way ANOVA (**C** and **D**).

The success of cancer immunotherapies, such as adoptive cell transfer, is highly dependent on the infiltration of T cells into the TME (19–21). Thus, we sought to determine the role of PD-1H in T cell tumor infiltration. We isolated OT-1 cells from CD45.2-WT or CD45.2-PK mice, we then transferred those OT-I cells into congenic CD45.1 mice, who — 6 days prior — had been inoculated with EG7 tumor cells (Figure 5A). Four days after adoptive transfer, tumors were harvested and evaluated for the presence of tumor-infiltrating cells. We found increased portions of total and donor CD8^+^ T cells within the TME of the PKO cell–transferred mice, while infiltration of host CD8^+^ T cells did not differ between the groups (Figure 5A). Additionally, we examined the chemotactic ability of activated T cells using a transwell migration assay in which EG7 culture supernatant was used as the chemoattractant. Compared with WT OT-I cells, PKO cells exhibited increased migratory activity (Figure 5B). These data suggest that abolishing intrinsic PD-1H enhances chemotactic migration of CD8^+^ T cells and promotes T cell infiltration into the TME. T cell trafficking is regulated by chemokines and chemokine receptors. The expression of chemokines and their receptors on WT OT-I and PKO cells was evaluated by quantitative PCR (qPCR) and flow cytometry. PKO cells exhibited significantly upregulated mRNA of *Cxcr3* and *Cxcr5* compared with WT OT-I cells while *Cxcr2*, -*4*, and -*6*; *Cx3cr1*; and *S1pr* remained less changed. Expression of chemokine mRNA revealed significant decreases of *Ccr1* and *Ccr8*, whereas other chemokines were less affected (Figure 5C). Expression of cell surface CXCR3 was also upregulated in a small but persistent level by flow cytometry analysis (Figure 5D). These results suggest a possible role of PD-1H in the regulation of chemokines and their receptors in T cells to modify their trafficking.

### PD-1H ablation promotes the effector function of human CAR-T cells.

**Figure 6:**
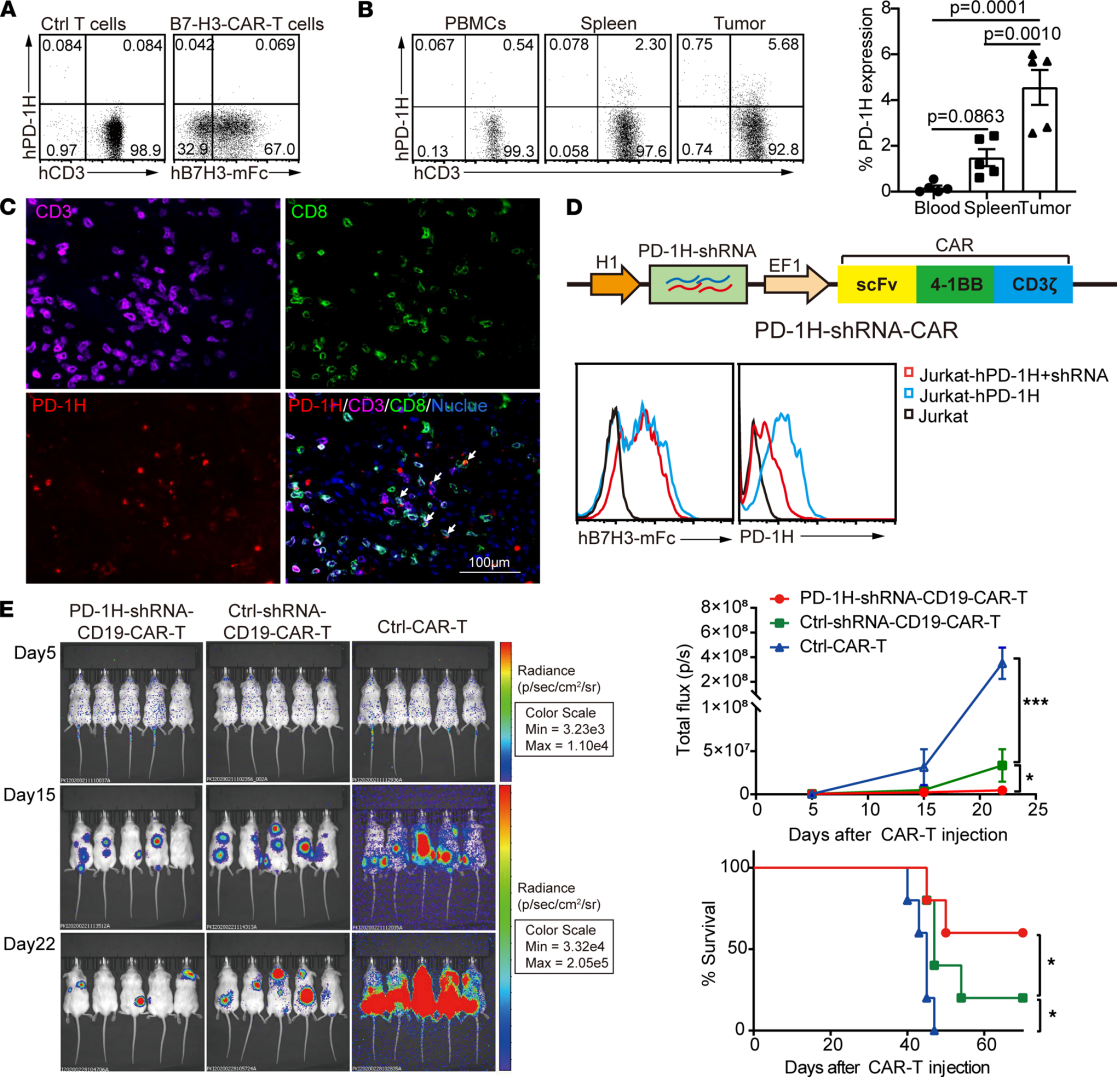
PD-1H ablation promotes the effector function of human CAR-T cells. B7-H3–specific CAR-T cells were generated using human PBMCs and were injected into NCG mice bearing colon PDX tumors. (**A**–**C**) Flow cytometry was used to evaluate PD-1H expression in the CAR-T cells generated in vitro (**A**), in human T cells isolated from peripheral blood of the mice (**B**), or presented in the tissues of PDX tumors (arrows) (**C**) after 25 days injection. Data presented as mean ± SEM. *P* values by 1-way ANOVA. (**D**) Schematic structure of PD-1H–shRNA-CAR coexpressing PD-1H targeting shRNA, and its efficiency in Jurkat-PD-1H cells. (**E**) BLI illustrating tumor growth in the CA-46 lymphoma mouse model. NCG mice were i.v. injected with 5 × 10^4^ firefly luciferase expressing CA-46 cells and randomly grouped (*n* = 5 per group). Five days after injection, mice were treated with 2 i.v. injections of 5 × 10^6^ indicated CAR-T Cells. Tumor growth was assessed by BLI on days 5, 15, and 22. The Kaplan Meier survival was analyzed. *P* values by log-rank (Mantel-Cox) test. *P < 0.05; ***P < 0.001.

With the effect of PD-1H on mouse CD8^+^ T cells in vivo, we tested if PD-1H plays a similar role in human T cells and if targeting PD-1H can improve the efficacy of adoptive CAR-T cell immunotherapy. PD-1H was not detectable by flow cytometry analysis in human T cells purified from healthy donor peripheral blood mononuclear cells (PBMCs) or B7-H3–specific CAR-T cells (22) (Figure 6A). Strikingly, when B7-H3–CAR-T cells were injected into NCG mice bearing s.c. patient-derived xenograft (PDX) tumors of colon cancer, PD-1H expression was upregulated in human T cells that had infiltrated the spleen and tumor tissues, whereas PD-1H expression in T cells from blood remained low (Figure 6, B and C). These findings suggest that PD-1H expression is induced in tissue-infiltrated human CAR-T cells and may regulate CAR-T cell function within the TME.

We next engineered the CAR-T cells that coexpressed a shRNA to knock down PD-1H (Figure 6D). Jurkat cells overexpressing PD-1H protein were utilized as positive controls to screen and identify shRNA sequences that are effective in knocking down PD-1H. Once a sequence was identified, we coexpressed that shRNA in the CD19-specific CAR-T cells and found that it efficiently downregulated PD-1H expression without interfering with CAR expression in human T cells (Figure 6D).

Finally, a suboptimal dose (1 × 10^6^ cells per mouse) of these CD19-CAR-T cells expressing the PD-1H targeted shRNA (PD-1H–shRNA–CD19–CAR-T cells) were used to treat NCG mice bearing CD19^+^ CA-46 lymphoma. Compared with CD19-CAR-T cells, the PD-1H–shRNA–CD19–CAR-T cells exhibited superior antitumor activity, which was indicated by prolonged survival of tumor-bearing mice. As a control, nontargeted CAR-T cells (B7-H3–CAR-T) cells were unable to suppress leukemia growth (Figure 6E). These results indicate that downregulation of PD-1H of CAR-T cells may improve their antitumor activity.

## Discussion

We demonstrate here that PD-1H is upregulated in CD8^+^ TILs and that ablation by either homologous recombination or, more practically, gene editing by CRISPR/Cas9 technology improves CD8^+^ T cell functionality — namely, cell proliferation and cytokine production. In addition, we further showed that deletion of PD-1H enhances cytotoxicity of activated T cells for target cells, and it enhances chemotactic activity to promote the migration. Importantly, silencing of PD-1H by gene editing enhanced the ability of mouse CD8^+^ T cells to suppress established tumors in syngeneic mouse models. To extend these findings, we engineered human CAR-T cells in which PD-1H was downregulated and showed its superior antitumor activity in a xenograft mouse model of human lymphoma. Therefore, upregulated PD-1H on T cells may be a target for manipulation to improve adoptive cell therapy.

The expression level of PD-1H varies among different subsets of immune cells. It has been shown that PD-1H is constitutively expressed in mouse T cells and myeloid cells in naive status (1, 2), whereas PD-1H disappears rapidly from the cell surface in culture (2, 4). In contrast, PD-1H is largely undetectable in human peripheral blood T cells (4). These findings cast doubt that, if PD-1H plays a role in the effector phase of T cells. Our studies reveal that cell surface PD-1H is upregulated in TILs in mouse tumor models and both CD4^+^ and CD8^+^ TILs maintain PD-1H expression. These data suggest that loss of PD-1H expression in the culture may be due to an in vitro culture condition that may not occur in vivo. We further show that PD-1H expression in vitro could be maintained in high cell density, supporting this possibility. PD-1H has been shown to suppress T cell activation via PSGL-1 in an acidic environment (11). In this context, we assessed whether acidic environments also altered PD-1H expression on T cells. In a range of pH 5.8–7.4, activated T cells did not change PD-1H expression levels in both mouse and human T cells (unpublished observations). Thus, the acidity of the TME may not be an inducer of PD-1H expression. Since the loss of PD-1H from cell surface upon activation occurs rapidly, often in several hours, it is less likely that this is due to the downregulation of PD-1H gene expression rather than the events on cell surface protein, which include but are not limited to internalization, shedding from cell surface, and the cleavage by specific proteases.

Physiological functions of PD-1H on T cells have been explored recently. PD-1H is constitutively expressed on naive T cells. Genetic ablation of PD-1H in mice, albeit without spontaneous activation of autoreactive T cells and autoimmune diseases, led to enhanced T cell responses to antigens, as well as to promotion of autoimmune diseases in mouse models (5, 7). Therefore, physiological function of PD-1H appears to keep T cells in check to prevent overreactivity of T cells to antigens, and this is consistent with a recent finding that PD-1H plays an essential role in keeping CD4^+^ T cells in a quiescent status (9). The upregulation of PD-1H on activated T cells and enhanced CD8^+^ T cell responses by ablating PD-1H as shown in our study would suggest a role of PD-1H to fine-tune T cell responses.

In this study, we specifically examined the effect of PD-1H on CD8^+^ T cells and showed that PK CD8^+^ T cells produced more IFN-γ and IL-10 than WT CD8^+^ T cells in response to antigen stimulation. In addition, ablation of PD-1H promoted effector functions of CD8^+^ T cells, including their capacities to lyse tumor cells. Finally, loss of PD-1H on T cells may also promote their ability to migrate into tumors, as shown in in vitro T cell chemotaxis assays and their increased infiltration of CD8^+^ T cells in the tumors. These findings are consistent to upregulated expression of several chemokine receptors, including CXCR3 and CXCR5. CD8^+^ T cells with CXCR3 and CXCR5 expression are shown to be highly functional subsets with capacities for spatial distribution, infiltration into tumors, and effector function (23–27). The expression of some chemokines by CD8^+^ T cells was also altered upon ablation of PD-1H in our study (Figure 5C). Since chemokines could be released by various types of cells in addition to T cells, the functional significance of these findings is less known. More importantly, the adoptive transfer of PK CD8^+^ T cells has improved cytotoxicity to inhibit established tumors in syngeneic mice, accompanied with an increased infiltration of T cells. Therefore, ablation of PD-1H may operate via multiple mechanisms to improve antitumor immunity. Our findings indicate that T cell–associated PD-1H downregulates the antitumor activity and that ablation of PD-1H could promote functionality and enhance therapeutic effect of T cells.

Given that inhibitory activity of PD-1H in T cells and ablation of PD-1H may improve the efficacy of T cell–mediated antitumor immunotherapies, we further extended this approach to humanized mouse models with adoptive transfer of CAR-engineered human T cells against human cancer. Though PD-1H is not detectable on the T cells from freshly isolated PBMCs and CAR-T cells generated in vitro, the expression level of PD-1H was substantially increased when the CAR-T cells infiltrated to TME and spleen, while no such increase was observed in the peripheral CAR-T cells. In a xenograft NCG model of CD19^+^ CA-46 lymphoma, CD19–CAR-T cells with shRNA-mediated ablation of PD-1H significantly improved the antitumor activity of the CAR-T cells to inhibit the lymphoma growth and prolonged the survival of tumor-bearing mice. Therefore, our results support the use of PD-1H–edited CAR-T cells to improve the therapeutic effect of CAR-T cells for cancer treatment.

## Methods

### Mice.

Six- to 8-week-old female C57BL/6 (B6) mice were purchased from Beijing Vital River Laboratory Animal Technology Company. PK mice on B6 background have been described previously (5). CD45.1 and OT-I mice were purchased from Nanjing Biomedical Research Institute of Nanjing University. OT-I mice were backcrossed to PK mice to generate PKO mice. All mice were housed in specific pathogen–free facilities and treated following IACUC standards of Fujian Medical University.

### Cell lines and cell culture.

Murine B16-F10, EG7, NIH/3T3, and CHO cell lines and human Jurkat, 293T, and CA-46 cell lines were purchased from ATCC. The B16-OVA cell line expressing OVA was generated from B16-F10. All cell lines were cultured in RPMI 1640 (Corning) and used within 5 passages for the experiments in this study. RPMI 1640 was supplemented with 10% heat-inactivated FBS (Thermo Fisher Scientific), 10 mM HEPES, 1 mM sodium pyruvate (Thermo Fisher Scientific, 11360-070), 2 mmol/L l-glutamine (Corning), 10 mmol/L HEPES (Corning), 1 U/mL penicillin, and 1 μg/mL streptomycin (Corning). The cell lines were tested to be Mycoplasma free by using Myco-Blue Mycoplasma Detector (Vazyme Biotech Ltd.).

### Peptides, antibodies, and flow cytometry analysis.

OVA_257–264_ (SIINFEKL, H-2K^b^) peptides were synthesized by Invitrogen. Antibodies used for flow cytometry analysis were purchased from eBioscience: anti-CD3 (clone 145-2C11), anti-CD4 (clone RM4-5), anti-CD8 (clone 53-6.7), anti-CD11c (clone N418), anti-CD11b (clone M1/70), anti–Gr-1 (clone RB6-8C5), anti–IFN-γ (clone XMG1.2), anti–granzyme B (clone NGZB), anti–TNF-α (clone MP6-XT22), anti-CD45.1 (clone A20), anti-CD45.2 (clone 104), anti–PD-1 (clone J43), anti-TOX (clone TXRX10), anti-LAG3 (clone C9B7W), anti-2B4 (clone eBio244F4), anti-TIM3 (clone RMT3-23), anti–T-bet (clone 4B10), anti-Eomes (clone Dan11mag), and anti–Bcl-2 (clone Bcl-2/100). The following antibodies were purchased from BioLegend: anti–PD-1H (clone MH5A), anti–hamster IgG (clone eBio299Arm), and anti-TIGIT (clone Vstm3). The following antibody was purchased from BD Biosciences: anti-TCF1 (clone S33-966). Intracellular cytokines staining was performed according to the manufacturer’s protocol of the Cytofix/Cytoperm Kit (BD Biosciences). Fluorescence was detected by flow cytometry (BD FACSVerse) and analyzed using FlowJo software (Tree Star Inc.).

### Tissue and blood samples.

Formalin-fixed paraffin-embedded or surgical tissue samples were obtained from the Department of Pathology, Union Hospital of Fujian Medical University. Informed consent was obtained before sample collection. Buffy coat collected from healthy adult volunteers was obtained from Fujian Blood Center under consent and was used to isolate PBMCs.

### T cell function analysis in vitro.

Naive WT or PK CD8^+^ T cells were isolated from mice using a CD8^+^ T cells isolation kit (Stemcell Technologies) and labeled with 1 μM CFSE (Invitrogen). CD8^+^ T cells were activated with anti-CD3 mAb or OVA peptide in the presence of irradiated PK APCs. Proliferation was assayed after 72 hours of activation by CFSE dilution. Cytokine analysis was performed using the mouse Th1/Th2/Th17 CBA kits (BD Bioscience).

In vitro, OVA-specific T cell response was measured using the CFSE/PI-labeling cytotoxicity assay. In brief, naive CD8^+^ T cells from WT OT-I and PKO mice were activated by OVA peptide and irradiated APCs for 7–15 days. Target tumor cells were labeled with 3 μM CFSE. Activated CD8^+^ T cells were cocultured with the target tumor cells at a different ratio of effector to target for 4 hours. After incubation, cells were stained with 0.1 μg DAPI (Sigma-Aldrich) and analyzed by flow cytometry.

### Murine tumor models.

B16-OVA (0.8 × 10^6^) or EG7 (0.4 × 10^6^) tumor cells were inoculated in the right flank of female mice on day 0, and tumor size was measured every other day using a digital caliper from day 5. The maximum tumor size allowed adhered to 20 mm in diameter. B16-OVA cell–bearing mice were transferred with 1 × 10^6^ (for EG7 model) or 2 × 10^6^ (for B16-OVA model) activated WT OT-I or PKO cells by i.v. injection at the fifth day following tumor cell inoculation when tumors were palpable or 5 mm. PBS was administered to the control mice.

For the analysis of TILs, tumors were typically harvested when the control tumors reached a diameter of 15–20 mm. Tumors were digested by Collagenase IV (0.2 mg/mL, Sigma-Aldrich) and DNase I (0.02 mg/mL, Sigma-Aldrich) for 30 minutes at 37°C. Tumor tissues were mashed with a syringe through 70 μm cell strainers. TILs were enriched using a 40%–80% Percoll (Sigma-Aldrich) gradient centrifugation (run 800*g* for 20 minutes at room temperature without brake) and harvested from the middle layer of the gradient. TILs were analyzed directly by flow cytometry based on markers of CD45, CD3, CD11b, Gr1, CD11c, CD4, and CD8.

For cytokine analysis of TILs in the B16-OVA tumor, TILs were restimulated by irradiated tumor cells overnight or with OVA peptide for 5 hours in 1640 medium with 10% FBS, 2 mM L-glutamine, 1% penicillin-streptavidin, 1 mM nonessential amino acids, mouse IL-2 (5 U/mL, Peprotech), and 1× Protein Transport Inhibitor Cocktail (eBioscience). The levels of cytokines IFN-γ and granzyme B were analyzed by intracellular staining and flow cytometry.

### Plasmid expression vectors.

The Cas9 expression construct LentiCRISPR v2 (Addgene 52961) was modified by adding an GFP sequence to replace the puro sequence (LentiCRISPR-GFP). Oligos for generation of sgRNA expression plasmids were annealed and cloned into the BsmBI sites of LentiCRISPR-GFP vector. pLKO.3G plasmid (Addgene 14748) was used for shRNA expression. The sgRNA sequences include the following: *Pdcd1*, 5′-CAACGTGGTTTGAGCCAACCCGTCC-3′; *Vsir*, PD-1H–1, 5′-CACCGATGTGAAGTTGCGTATGGGC-3′; PD-1H–2, 5′-CACCGACAACGGTTCTACGGGTCCA-3′. The shRNA sequences include the following: *Pdcd1*, 5′-AATTGACATGAGGATGGACATTGTTCTCGAGAACAATGTCCATCCTCATGTCTTTTTTTAT-3′; *VSIR*, 5′-CCGGCCCTGACTCTCCAAACTTTGACTCGAGTCAAAGTTTGGAGAGTCAGGGTTTTTG-3′; control shRNA, 5′-CCGGTCGCCATCAGTCGTACTTGATCTCGAGATCAAGTACGACTGATGGCGATTTTTG-3′.

### Lentivirus production and infection.

Lentivirus was produced by cotransfection of the LentiCRISPR-GFP vector with the packaging plasmids psPAX2 and pCMV-VSV-G (Addgene, 12260 and 8454) into 293T cells using polyethylenimine (PEI, Sigma-Aldrich) transfection. For each flask, 5 μg of lentiviral transfer vector, 3 μg of psPAX2, and 2 μg pCMV-VSV-G were diluted in 500 μL OptiMEM (Invitrogen). PEI of 30 μg was diluted in 500 μL OptiMEM 5 minutes before it was added to the mixture of DNA and PEI. The complete mixture was incubated for 15 minutes before added to cells. After 8 hours, the medium was changed to 15 mL DMEM supplemented with 10% FBS. The virus-containing supernatant was collected at 48 hours and 72 hours after transfection and centrifuged at 1200*g* at 4°C for 10 minutes to pellet cell debris. The supernatant was filtered through a 0.45 μm filter (MilliporeSigma) and concentrated by ultracentrifuging at 100,000*g* for 2 hours at 4°C. Subsequently, the lentivirus particles were resuspended in an appropriate volume using RPMI-1640 medium at 4°C overnight. Final viral samples were titrated and stored at −80°C in single-use aliquots.

### CAR-T cell generation.

Human PBMCs were transduced with lentivirus particles of corresponding CAR-expressing plasmids, as described in a previous study (22). In brief, Human PBMCs from healthy donors were purified and then activated by culture with anti-CD3/CD28 beads (Thermo Fisher Scientific) according to the manufacturer’s protocol. Two days after activation, PBMCs were transduced with indicated lentivirus particles. Spinoculation was performed for 2 hours at 900*g*, 32°C, followed by incubation for 48 hours. After that, cells were pelleted and resuspended in the fresh media containing IL-2 (1 × 10^5^ U/mL). Cells were counted and fed every 2 days. Transduction efficiencies were analyzed by flow cytometry to confirm the generation of CAR-T cells.

### T7EN1 cleavage assay and sequencing.

NIH/3T3 cells were transfected with sgRNA LentiCRISPR-GFP vector using Lipofectamine 2000 (Invitrogen) following the manufacturer’s protocol. Cells were incubated at 37°C for 72 hours after transfection before genomic DNA extraction. In total, 0.05 × 10^6^ to 0.2 × 10^6^ cells were resuspended in 100 μL of QuickExtract solution (Epicentre), which was added to lyse the cells, and the genomic DNA was extracted following the manufacturer’s protocol. The genomic regions, containing the target sites of PD-1H, were PCR amplified, and products were purified using QiaQuick Spin Column (Qiagen) following the manufacturer’s protocol. The primers are as follows: PD-1H forward, 5′-GTCTGGCTTGCAAGGGAAGAGGC-3′ and PD-1H reverse, 5′-CAAGATAATCACACGTCTGCAC-3′.

The levels of genomic disruption of PD-1H in NIH/3T3 cells or T cells were determined by the T7EN1 Nuclease assay (NEB). Hybridized PCR products in 200 ng were digested with T7EN1 for 20 minutes and analyzed on 10% polyacrylamide TBE gel. Gels were stained with SYBR Safe DNA stain (Invitrogen) for 15 minutes and imaged using a Gel Doc gel imaging system (Bio-Rad). The DNA band intensity was quantitated using ImageJ (NIH). Indel percentage was determined by the formula, 100 × (1 – [1 – (b + c)/(a + b + c)]^1/2^), where a is the band intensity of the undigested PCR product, and b and c are the band intensity of each cleavage product. The PCR products were also sequenced for TIDE analyses (tide.nki.nl). All TIDE analyses below the detection sensitivity of 1.5% were set to 0%. The purified PCR products were ligated with the pMD18-T vector using the pMD18-T Vector Cloning Kit (Takara) to detect mutant alleles. Ligation products were used for transformation, and about 20–30 colonies per sample are sequenced using universal primer M13F. The top 5 targets for each sgRNA were amplified by PCR, as described previously, and subjected to Sanger sequencing. Sequencing results were analyzed using the TIDE method. Primers used for PCR are listed: OT-1, 5′-GTGACCAGGATCTAAGGGTGC-3′ and 5′-GCCTGCTCCAAGGAGCTTGGT-3′; OT-2, 5′-ACCCTGGCCACATTTCAGTTG-3′ and 5′-CAGCTGGAAGGTTGGCATT-3′; OT-3, 5′-GCTTCATATGCACTTGGCCT-3′ and 5′-CACTTAGTCCCTACTTTACCT-3′; OT-4, 5′-ATCATGGCCTCCTTCTCGGC-3′ and 5′-TCTATAATTGAGCCCTGCTCC-3′; and OT-5, 5′-ATCTGGGAACTGAATGATTAG-3′ and 5′-GCTGCTTCTGTTCATCGTGGC-3′.

### qPCR.

Total RNA was prepared using the Total RNA Kit I (Omega Bio-tek) according to the manufacturer’s protocol. cDNA was made using the PrimeScript RT reagent Kit with gDNA Eraser (Takara), and gene expression was detected using the SYBR Premix Ex Taq II (Takara). Relative quantification was calculated using the 2^–ΔΔCt^ method. Data were normalized to reference gene β-actin. The gene-specific primers are listed: *Vsir* forward, 5′-GGCCCATACGCAACTTCACAT-3′; *Vsir* reverse, 5′-TGGACCCGTAGAACCGTTGT-3′; *Pdcd1* forward, 5′-ACCCTGGTCATTCACTTGGG-3′; *Pdcd1* reverse, 5′-CATTTGCTCCCTCTGACACTG-3′; *b-actin* forward, 5′-GATTACTGCTCTGGCTCCTAGC-3′; *b-actin* reverse, 5′-GACTCATCGTACTCCTGCTTGC-3′; *Ccr1* forward, 5′-CTCATGCAGCATAGGAGGCTT-3′; *Ccr1* reverse, 5′-AGCTTGCACATGGCATCACC-3′; *Ccr2* forward, 5′-ATCCACGGCATACTATCAACATC-3′; *Ccr2* reverse, 5′-CAAGGCTCACCATCATCGTAG-3′; *Ccr3* forward, 5′-TCGAGCCCGAACTGTGACT-3′; *Ccr3* reverse, 5′-CCTCTGGATAGCGAGGACTG-3′; *Ccr5* forward, 5′-GTCAGTTCCGACCTATAGC-3′; *Ccr5* reverse, 5′-TGCAGCTTATCAAGATGAG-3′; *Ccr7* forward, 5′-TGTACGAGTCGGTGTGCTTC-3′; *Ccr7* reverse, 5′-GGTAGGTATCCGTCATGGTCTTG-3′; *Ccr8* forward, 5′-ACGTCACGATGACCGACTACT-3′; *Ccr8* reverse, 5′-CGAGGACTAAGATGACCAGG-3′; *Cxcr2* forward, 5′-ATGCCCTCTATTCTGCCAGAT-3′; *Cxcr2* reverse, 5′-GTGCTCCGGTTGTATAAGATGAC-3′; *Cxcr3* forward, 5′-GGTTAGTGAACGTCAAGTGCT-3′; *Cxcr3* reverse, 5′-CCATAATCGTAGGGAGAGGT-3′; *Cxcr4* forward, 5′-CTTCTGGGCAGTTGATGCCAT-3′; *Cxcr4* reverse, 5′-CTGTTGGTGGCGTGGACAAT-3′; *Cxcr5* forward, 5′-TGGCCTTCTACAGTAACAGCA-3′; *Cxcr5* reverse, 5′-GCATGAATACCGCCTTAAAGGAC-3′; *Cxcr6* forward, 5′-GAGTCAGCTCTGTACGATGG-3′; *Cxcr6* reverse, 5′-TCCTTGAACTTTAGGAAGCG-3′; *Cx3cr1* forward, 5′-AGTATGACGATTCTGCTGAGG-3′; *Cx3cr1* reverse, 5′-CAGACCGAACGTGAAGACGAG-3′; *S1pr* forward, 5′-ATGGTGTCCACTAGCATCC-3′; *S1pr* reverse, 5′-CGATGTTCAACTTGCCTGTG-3′.

### T cell migration studies.

CD45.1^+^ congenic mice were inoculated with EG7 tumor and were i.v. injected with purified CD45.2^+^ WT OT-I or PKO cells on day 6. The tumor was harvested and analyzed on day 10. TILs were analyzed directly by flow cytometry based on markers CD45.1, CD45.2, CD3, CD4, and CD8. In vitro migration assay was performed in media containing 0.5% FBS. In total, 0.3 × 10^6^ activated WT OT-I or PKO cells were applied to the upper chamber of 24-well plate transwell with a pore size of 5 μm (Corning). Lower chambers were set up with EG7 culture supernatant or with 1640 medium. Plates were incubated for 3.5 hours, and then the cell number of lower chambers was counted. Each condition was performed in triple wells.

### PDX mouse models and treatment.

Eight- to 10-week-old female NCG mice (NOD-Prkdcem26Cd52Il2rgem26Cd22/Nju) were used for xenograft model generation. All experiments with the immunodeficient mice were performed following the guidelines approved by the IACUC of Fujian Medical University. The patient’s colon cancer tissues were cut into fragments of approximately 15 mm^3^ by surgery and implanted s.c. into the bilateral axilla of female NCG mice within 2 hours after surgery. The implanted mice were observed daily until 90 days. Tumors were measured once a week by caliper. The xenografted tumors (~500 mm^3^) subsequently harvested from the mice were further implanted and expanded in NCG mice. After 5 consecutive mouse-to-mouse passages, the xenografted tumors were implanted into the bilateral axilla of NCG mice. When the tumors reached a mean diameter of approximately 8 mm, mice were i.v. injected with 3 × 10^6^ CAR-T cells or i.t. injected with 2 × 10^6^ CAR-T cells, respectively. On day 25, mice were sacrificed, the tissues and blood samples were obtained, and single-cell suspensions were analyzed by flow cytometry.

### Multiplex immunofluorescence.

Freshly cut PDX sections were deparaffinized, and antigen retrieval was carried on with 1 mmol/L EDTA (pH 8, Maixin Biological Technology) and boiled for 20 minutes at 97°C. Inactivation of endogenous peroxidase activity was carried on using a solution of 0.3% hydrogen peroxide for 20 minutes, and then slides were incubated with a blocking solution containing 0.3% BSA in 0.05% Tween-20 and Tris-buffered solution for 30 minutes. Slides were incubated with the primary antibody (PD-1H, clone D1L2G, Cell Signaling Technology) at room temperature for 2 hours. Isotype-specific HRP–conjugated antibodies and tyramide-based amplification systems (TSA Plus Fluorescence Kits) were used for signal detection according to the manufacturer’s instruction. PD-1H was selectively measured in immune cell subpopulations defined by its colocalization with the immune cell phenotype markers CD3, CD4, and CD8, followed by incubation with the appropriate secondary antibodies coupled to Alexa Fluor 488 or Alexa Fluor 647 (Invitrogen) and counterstaining of the nuclei with DAPI (Sigma-Aldrich). Tumor tissue was scanned using the EVOS FL Auto Cell Imaging System (EVOS FL Auto, Invitrogen).

NCG mice were i.v. injected with 5 × 10^4^ CA-46 cells expressing firefly luciferase. The mice were then i.v. treated with 5 × 10^6^ CAR-T cells (PD-1H–shRNA–CD19–CAR-T, control-shRNA–CD19–CAR-T, and control-B7-H3–CAR-T) on day 5, 10, and 15. Tumor growth in each treatment group of mice (*n* = 5 per group) was assessed by bioluminescent imaging on days 5, 15, and 22. For the survival analysis, separated experiments were performed with the same treatments to the groups of mice, which consisted of *n* = 5 per group.

### Statistics.

GraphPad Prism 7.0 was used for all statistical analyses. The mean ± SEM was determined for each treatment group in the individual experiments. Two-tailed unpaired *t* test, 1-way ANOVA, or 2-way ANOVA was used to determine the statistical significance between groups. *P* values of less than 0.05 were considered significant.

### Study approval.

All mouse procedures and experiments were approved by the IACUC of Fujian Medical University. Use of samples from human subjects was approved by the Committee of Ethical Review of Research, Fujian Medical University, and informed consent was acquired.

## Author contributions

LH, GH, and Lieping Chen designed the study and wrote the manuscript. LH, ZX, XZ, and Ling Chen performed the experiments and analyzed data. YC and NX participated in designed and performed PDX mouse experiments. CC edited the manuscript. LL, GH, and Lieping Chen supervised the experiments.

## Supplementary Material

Supplemental data

## Version 1 12/14/2021

In-Press Preview

## Version 2 01/25/2022

Electronic publication
