# Supplementary material for: Ablation of T cell–associated PD-1H enhances functionality and promotes adoptive immunotherapy
Source: JCI Insight. 2022 Jan 25;7(2):e148247. doi: 10.1172/jci.insight.148247 (PMC8855794; doi:10.1172/jci.insight.148247)
Supplement: Supplemental data [file jciinsight-7-148247-s183.pdf]

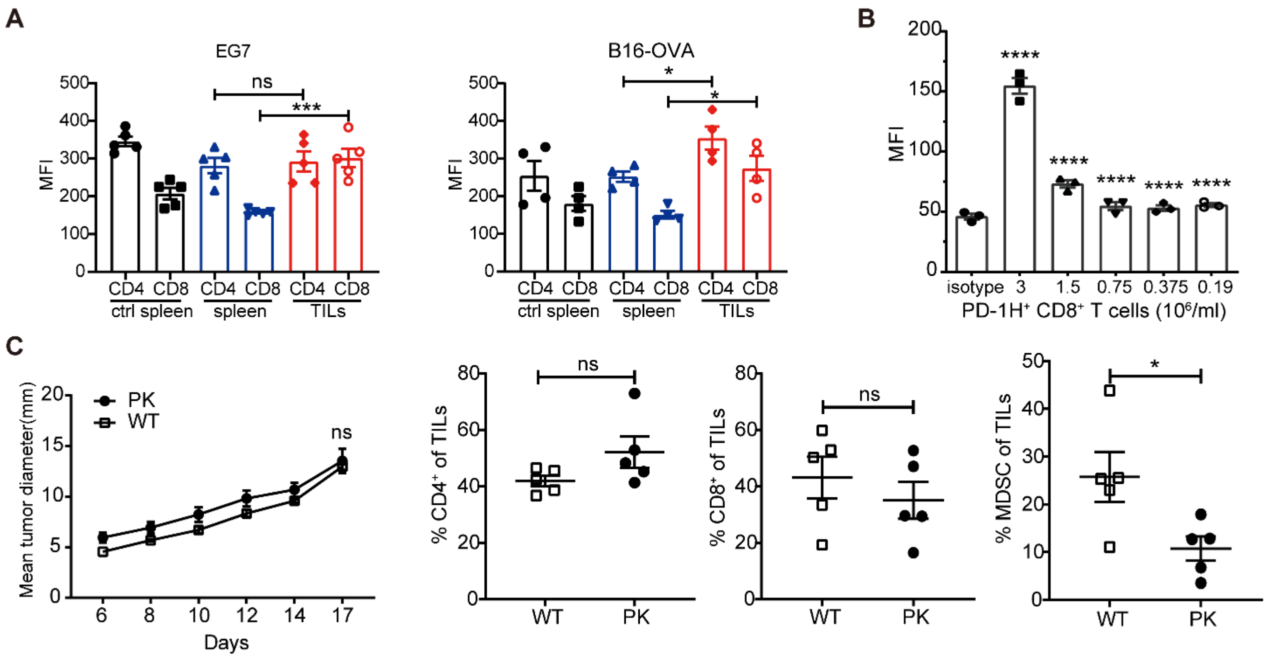

Figure S1

B16-OVA melanoma growth in WT and PK mice. (**A**) The median fluorescence intensity of PD-1H expression on EG7 and B16-OVA TILs, including $CD4^+$ and $CD8^+$ T cells. Data is representative of 3 independent experiments with mean ± SEM. ns, not significant. *P<0.05, ****P*<0.001 (two-tailed unpaired t-test). (**B**) The median fluorescence intensity of PD-1H expression on $CD8^+$ T cells in vitro culture for 72 h is shown. Data presented as mean ± SEM and representative 3 independent experiments. ****P<0.0001 (two-way ANOVA). (**C**) B16-OVA tumor cells were inoculated into the flank of WT and PK mice (n=5 per group). Tumors were harvested on day 17 post-tumor inoculation. TILs, including $CD4^+$ and $CD8^+$ T cells, and MDSCs ($CD11b^+$ $CD11c^-$ $Gr\text{-}1^+$) were analyzed by flow cytometry. Data are representative of 2 independent experiments. Mean ± SEM is shown and analyzed with a two-tailed unpaired *t*-test. **P*<0.05; ns, not significant.

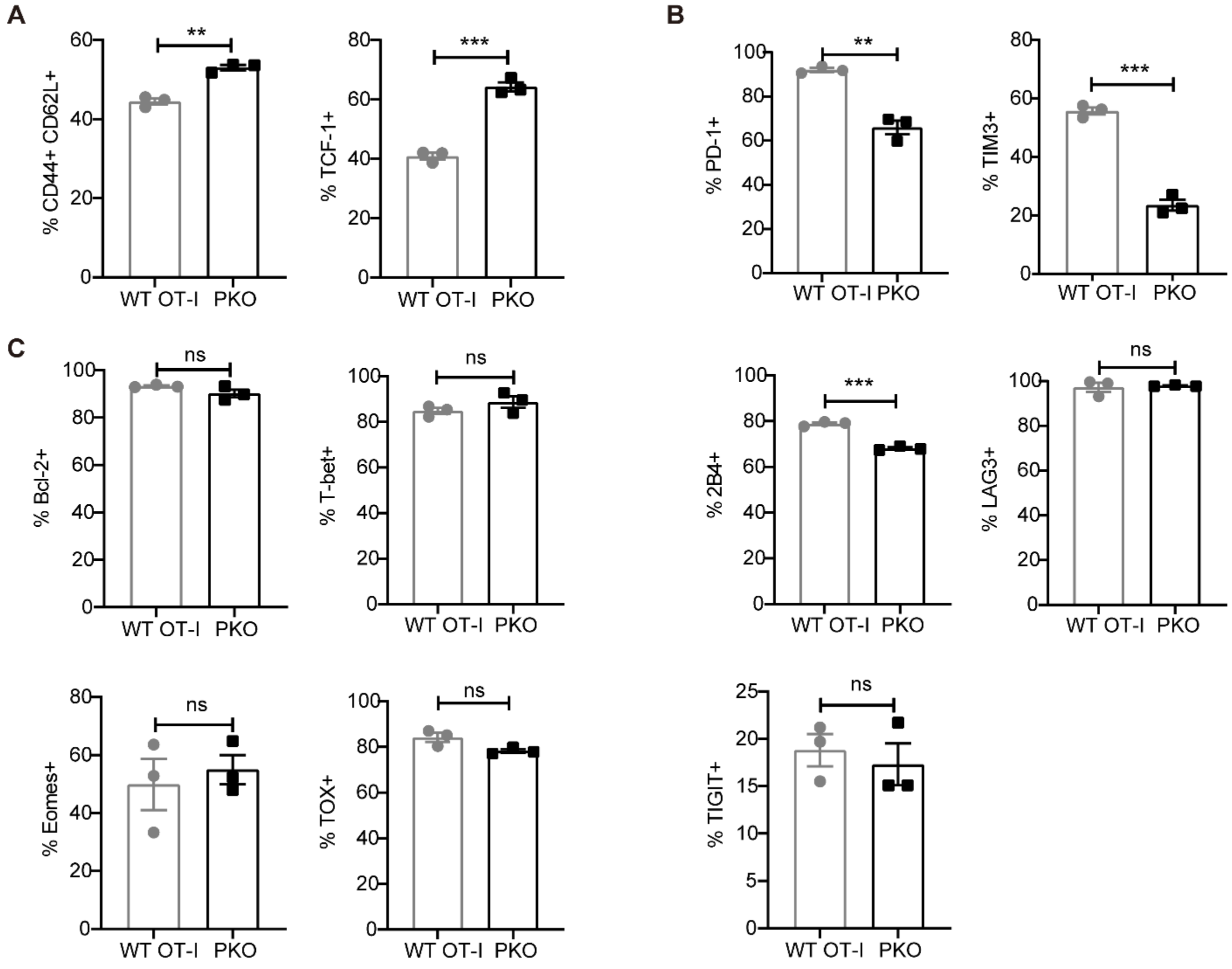

Figure S2

The phenotypic characterization of PD-1H deficient CD8^{+} T cells. Purified of PKO or WT OT-I cells were stimulated with OVA peptide-pulsed irradiated splenocytes. The expression levels of memory markers (CD44, CD62L and TCF1) and exhaustion markers (PD-1, Tim3, 2B4, LAG3 and TIGIT) were analyzed by flow cytometry (**A and B**). Key transcription molecules (Eomes, T-bet and TOX) and survival molecules (Bcl-2) were analyzed by intracellular staining (**C**). Mean ± SEM is shown and analyzed with a two-tailed unpaired *t*-test. ***P*<0.01; ****P*<0.001; ns, nonsignificant. Data are representative of 3 independent experiments.

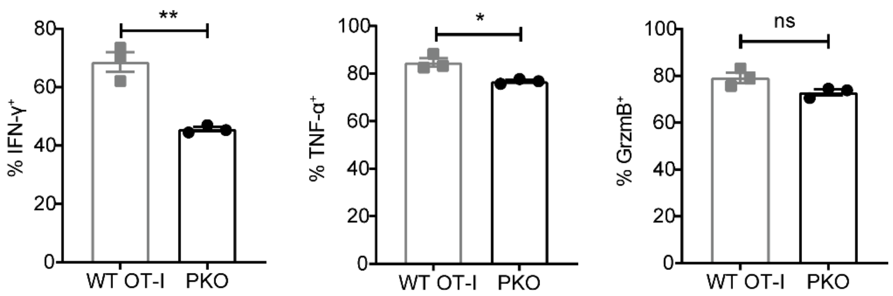

Figure S3

The expression level of effector molecules by OT-I cells. Activated PKO or WT OT-I cells were stimulated for 5 h with OVA peptide, and the production of IFN-γ, granzyme B, and TNF-α was analyzed by intracellular staining. Mean ± SEM is shown and analyzed with a two-tailed unpaired *t*-test. *P<0.05; **P<0.01; ns, nonsignificant. Data are representative of 3 independent experiments.

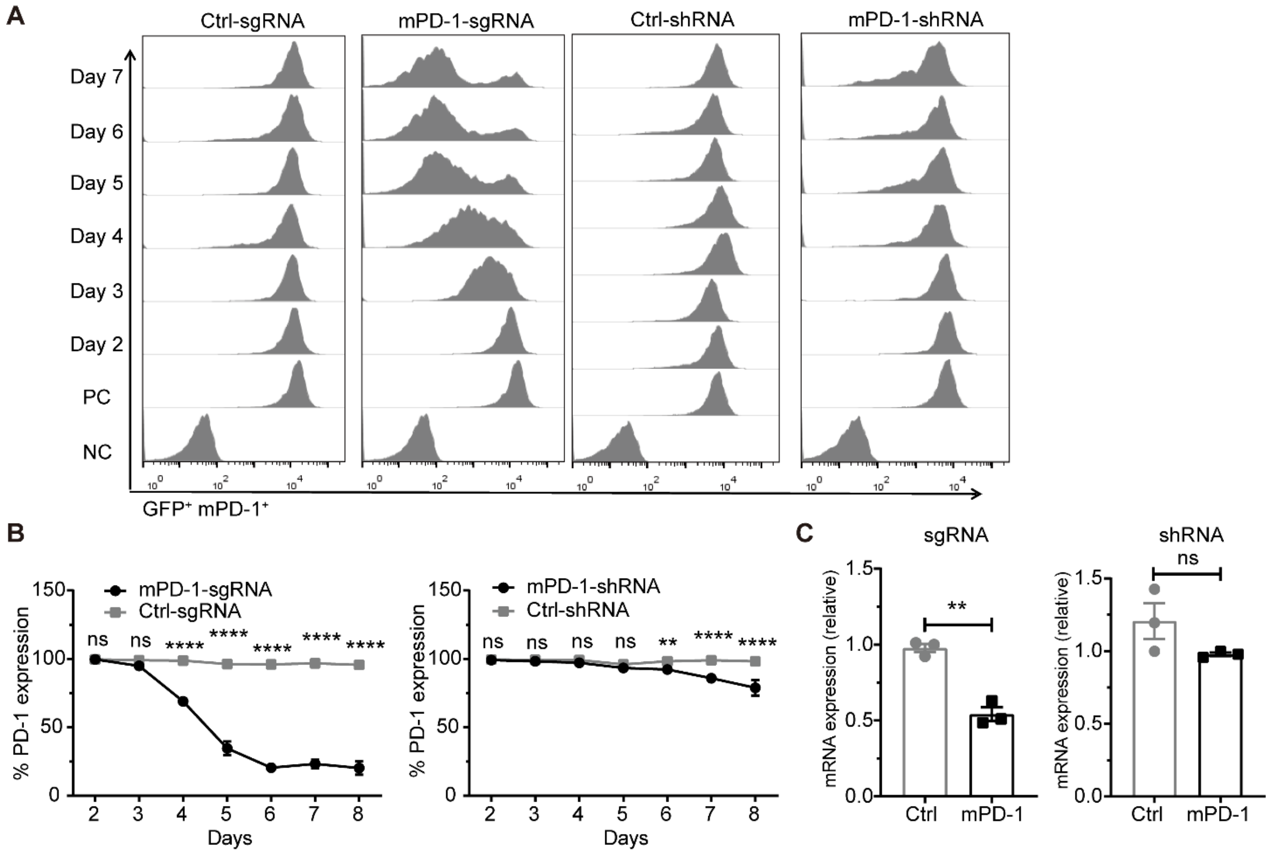

Figure S4

The disruption efficiency of the CRISPR/Cas9- and shRNA interference was compared on CHO/mPD-1 cell line. sgRNA or shRNA was transfected into CHO/mPD-1 cells. PD-1 expression was analyzed in the GFP^{+} gate by flow cytometry at a series of time points (**A** and **B**). Data is representative of 3 independent experiments with mean ± SEM. ns, not significant. $^{**}P<0.01$, $^{****}P<0.0001$ (two-way ANOVA). Collected CHO/mPD-1 cells on day 10 post-infection to quantify PD-1 mRNA expression by real-time PCR (**C**). Ctrl, negative control; P, positive control. Data are Mean ± SEM and representative of three independent experiments. ns, not significant. $^{**}P<0.01$ (unpaired *t*-test).

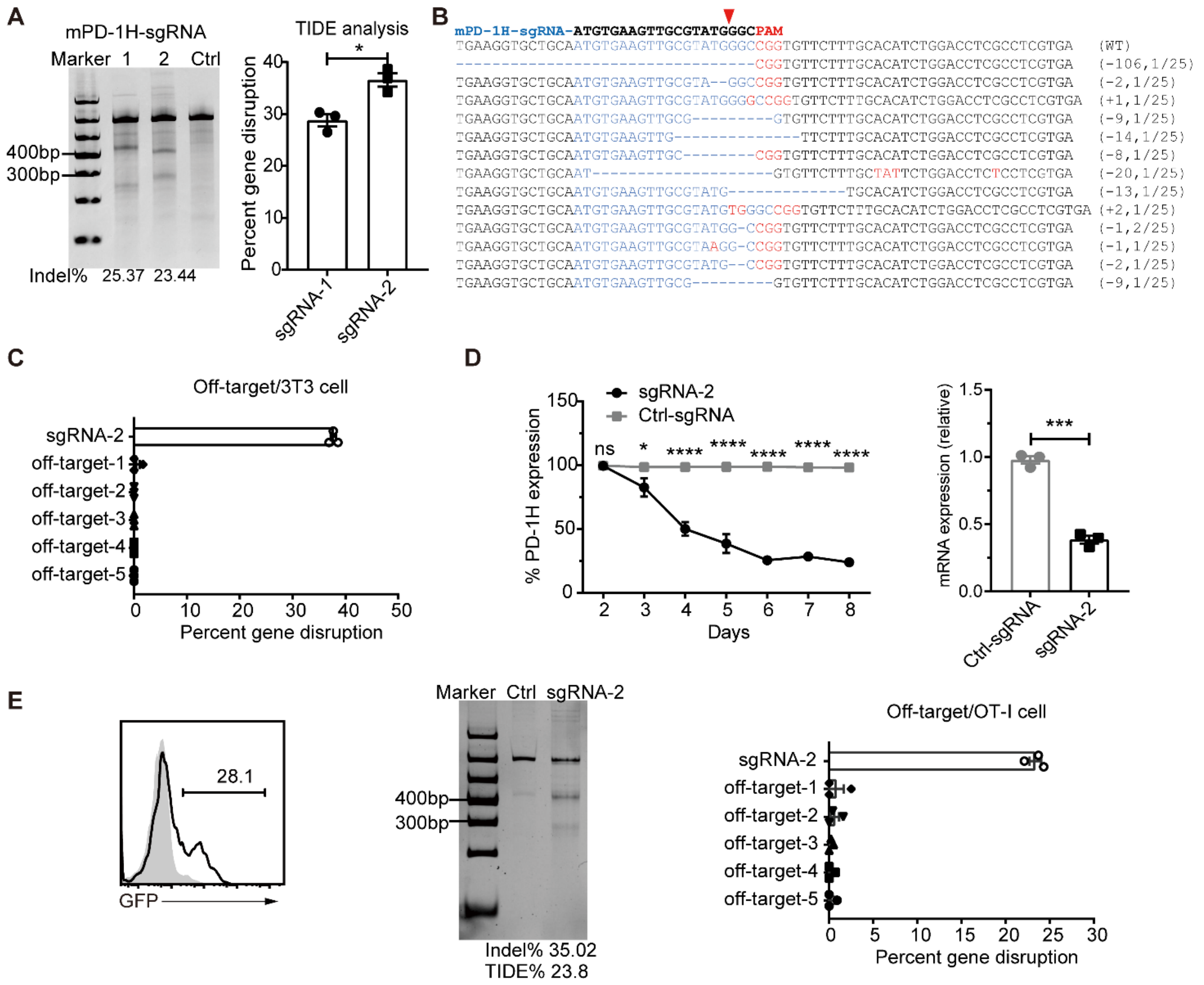

Figure S5

The design and validation of sgRNA targeting mouse PD-1H (mPD-1H). mPD-1H sgRNA were transfected into NIH/3T3 cells separately. NIH/3T3 cells were collected on day 3 post-transduction. Detection of sgRNA/Cas9-mediated cleavage of mPD-1H by T7EN1 cleavage assay and TIDE analysis (**A**). Data is representative of 3 independent experiments with mean ± SEM. *P<0.05 (unpaired t-test). Indels observed by clonal sequence analysis of PCR amplicons from the CRISPR-edited region in the gene expressing PD-1H (**B**). The red arrow indicates the putative cleavage site. The PAM sequences are highlighted in red and the targeting sequences in blue. Blue base or dot in the clonal sequences indicated insertion (+) or deletion (-) base, respectively. (**C**) Off-target

mutagenesis measurement of mPD-1H sgRNA on NIH/3T3 cells. sgRNA-2 disrupt PD-1H mRNA and cell surface expression on CHO/mPD-1H cells (**D**). Data is representative of 2 independent experiments with mean ± SEM. ns, not significant. *P<0.05, ***P<0.001, ****P<0.0001 by unpaired *t*-test and two-way ANOVA. CRISPR/Cas9 gene-editing components and sgRNA-2 delivered into mouse $CD8^+$ T cells by a lentiviral vector system. The percentage of GFP^+ indicated the efficiency of lentiviral transduction. Three days post transduction, gene-editing efficiency was confirmed by T7EN1 cleavage assay and by sequencing the mutation at the genomic locus of PD-1H (**E**). All TIDE analyses below the detection sensitivity of 1.5% were set to 0%. OT, off-target. Ctrl, negative control.
